# Effects of Stem Cell-Derived Mitochondrial Transfer on H_2_O_2_-Induced Oxidative Stress in COV434 Granulosa Cells

**DOI:** 10.3390/medicina62101951

**Published:** 2026-10-09

**Authors:** Selenay Furat, Candan Altuntaş, Kamil Can Kiliç

**Affiliations:** 1 Department of Stem Cell, Institute of Health Sciences, Kocaeli University, 41001 Kocaeli, Türkiye; 2 Center for Stem Cell and Gene Therapies Research and Practice, Kocaeli University, 41001 Kocaeli, Türkiye; 3 Department of Histology and Embryology, Faculty of Medicine, Kocaeli University, 41001 Kocaeli, Türkiye

**Keywords:** granulosa cells, mitochondrial transfer, mesenchymal stem cells, oxidative stress, apoptosis

## Abstract

*Background and Objectives*: Oxidative stress-induced mitochondrial dysfunction contributes to granulosa cell apoptosis and ovarian dysfunction. This study investigated the therapeutic potential of human Wharton’s jelly-derived mesenchymal stem cell-derived mitochondria (hWJ-MSCs-mito) in an H_2_O_2_-induced oxidative stress model of COV434 granulosa cells. *Materials and Methods*: Oxidative stress was induced using increasing concentrations of H_2_O_2_, and cell viability was assessed by the WST-8 assay. Isolated hWJ-MSC-mito were labeled with TMRM, transferred into COV434 cells, and confirmed by confocal microscopy. The expression of oxidative stress- and mitochondrial-related genes (*CYP19A1*, *GPX1*, *HO1*, *SOD1*, and *TOMM20*) was analyzed by qRT-PCR, whereas proliferative and apoptotic responses were evaluated by PCNA and active caspase-3 immunofluorescence, respectively. *Results*: Exposure to 1.5 mM H_2_O_2_ reduced cell viability by approximately 50%, establishing an oxidative stress model, while 10 µg hWJ-MSCs-mito provided an optimal mitochondrial dose. Transferred mitochondria were successfully internalized by recipient cells and enhanced cell viability, increased PCNA immunoreactivity, and reduced active caspase-3 immunoreactivity. Furthermore, it attenuated H_2_O_2_-induced upregulation of *CYP19A1* and HO1, moderately reduced SOD1 expression, preserved GPX1 expression, and showed a modest increase in TOMM20 expression. *Conclusions*: hWJ-MSC-mito supplementation was associated with improved cell viability, increased PCNA immunoreactivity, reduced active caspase-3 immunoreactivity, and modulation of selected transcriptional responses in H_2_O_2_-treated COV434 cells. These findings support further investigation of mitochondrial supplementation in oxidative stress-induced granulosa cell injury.

## 1. Introduction

Granulosa cells are specialized somatic cells that play a central role in steroid hormone synthesis and follicular development. Estradiol (E2), the predominant biologically active estrogen secreted by granulosa cells [1], is essential for oocyte quality and follicular survival [2]. Granulosa cells also support oocyte development, protect against oxidative stress, and contribute to reproductive function [3,4]. Conversely, granulosa cell apoptosis contributes to age-related follicular loss and represents a key mechanism underlying follicular atresia and premature ovarian insufficiency [5]. Oxidative stress-associated mitochondrial dysfunction and granulosa cell apoptosis have also been implicated in polyendocrine metabolic ovarian syndrome (PMOS). Alterations in granulosa cell function are associated with poor oocyte quality, impaired embryo development, and reduced pregnancy rates, while granulosa cell apoptosis is a key process underlying follicular atresia and female reproductive disorders. Apoptosis is regulated through mitochondrial (intrinsic) and death receptor (extrinsic) pathways [6]. Granulosa cells are particularly susceptible to ROS, especially H_2_O_2_, which can induce apoptosis [7]. Excessive ROS disrupts antioxidant defenses, leading to oxidative stress [8] and subsequent impairment of granulosa cell function, follicular development, and oocyte quality [9].

Mitochondria are major intracellular targets of oxidative stress in granulosa cells and are essential for steroid hormone biosynthesis, cellular bioenergetics, and metabolic homeostasis [10]. Oocytes rely on metabolites such as pyruvate and lactate generated by surrounding granulosa cells to meet the energy demands of growth and maturation [11,12], making granulosa cell mitochondrial integrity important for oocyte developmental potential. Mitochondria are also a major source of ROS [11,13], whose levels are normally regulated by antioxidant defenses and mitochondrial fission-fusion dynamics [13]. Excessive ROS disrupts this balance, leading to DNA damage, lipid peroxidation, impaired ETC activity, mitochondrial dysfunction, and activation of apoptotic pathways [14]. Accordingly, mitochondrial alterations in degenerating granulosa and cumulus cells have been associated with apoptosis and follicular atresia [15]. Thus, maintaining mitochondrial function is essential for granulosa cell survival, oocyte maturation, follicular development, fertilization, and preimplantation embryonic development [16].

Approaches aimed at preserving mitochondrial function have gained increasing attention in reproductive biology and regenerative medicine. Mesenchymal stem cells (MSCs), isolated from sources including bone marrow, umbilical cord, menstrual blood, endometrium, and adipose tissue [17], have regenerative and tissue-supportive properties. Among these, Wharton’s jelly-derived MSCs (WJ-MSCs) are particularly attractive due to their neonatal origin, ease of procurement, and enhanced antioxidant and anti-aging capacities [18]. Their self-renewal and multilineage differentiation capacities support their use in regenerative medicine [19,20], while preclinical and clinical studies have investigated their therapeutic potential in female reproductive disorders [21,22,23,24,25]. Evidence indicates that MSC effects are mediated largely through paracrine mechanisms rather than direct differentiation [26,27]. Importantly, mitochondrial transfer from MSCs to recipient cells has emerged as a mechanism of intercellular communication that may modulate cellular responses under stress conditions [28].

Mitochondrial transfer has emerged as a potential cellular repair mechanism underlying the beneficial effects of MSCs. Given the contribution of mitochondrial dysfunction to impaired granulosa cell function, transfer of healthy mitochondria to damaged cells has been proposed to support cellular bioenergetics and recovery. Mitochondrial transfer has been associated with enhanced survival and repair in several cell types; however, its effects in the female reproductive system remain poorly understood. In particular, evidence regarding mitochondrial transfer in granulosa cells exposed to oxidative stress is still limited. Accordingly, the present study aimed to investigate whether mitochondria isolated from human Wharton’s jelly-derived mesenchymal stem cells (hWJ-MSCs) could attenuate H_2_O_2_-induced oxidative injury in COV434 human granulosa cells by evaluating their effects on cell viability and apoptotic responses.

## 2. Materials and Methods

### 2.1. Cell Culture Procedures for COV434 Cells and hWJ-MSCs

In our study, the human granulosa cell line COV434 (Sigma-Aldrich, ECACC, Catalog Number: 07071909, St. Louis, MO, USA) was obtained from the cell collection of the Center for Stem Cell and Gene Therapies Research and Practice, Kocaeli University. The hWJ-MSCs used in the present study had been previously isolated and characterized in our earlier study [29] and were obtained from the cell collection of the Center for Stem Cell and Gene Therapies Research and Practice at Kocaeli University. Cells were cultured in Dulbecco’s Modified Eagle’s Medium/Nutrient Mixture F-12 Ham (DMEM/F-12; Sigma-Aldrich, Catalog Number: D9785, St. Louis, MO, USA) supplemented with 10% fetal bovine serum (FBS; Capricorn Scientific, Catalog Number: FBS-26A, Ebsdorfergrund, Hesse, Germany) and 1% penicillin-streptomycin (Pen/Strep, 10,000 U/mL; Sigma-Aldrich, Catalog Number: P4333, St. Louis, MO, USA). Cell cultures were maintained at 37 °C in a humidified atmosphere containing 5% CO_2_ under standard culture conditions. The culture medium was renewed every three days throughout the experimental period. Upon reaching approximately 70% confluency, cells were detached using 0.25% trypsin-EDTA (Capricorn Scientific, Catalog Number: TRY-3B, Ebsdorfergrund, Hesse, Germany) and subcultured into 25 cm^2^ tissue culture flasks at a split ratio of 1:4 to ensure continuous expansion and maintenance of the cell population for subsequent experiments.

### 2.2. Establishment of H_2_O_2_-Induced Oxidative Stress in COV434 Cells and Determination of the Optimal Dose Based on Cell Viability

To establish an H_2_O_2_-induced oxidative stress model in COV434 granulosa cells, cells were exposed to increasing concentrations of hydrogen peroxide (H_2_O_2_; Sigma-Aldrich, Catalog Number: 88597, St. Louis, MO, USA). For cell seeding, COV434 cells were detached using 0.25% trypsin-EDTA (Capricorn Scientific), collected into 50 mL Falcon tubes, and centrifuged at 90× *g* for 5 min at 4 °C. The supernatant was discarded, and the cell pellet was gently resuspended by pipetting 10–15 times to obtain a homogeneous single-cell suspension. Subsequently, 1 × 10^4^ cells were seeded into each well of 96-well culture plates. Following cell attachment, the culture medium was replaced with DMEM/F-12 containing H_2_O_2_ at final concentrations of 0.75, 1.5, 3, or 6 mM. Cells were exposed to H_2_O_2_ for 6 h at 37 °C in a humidified atmosphere containing 5% CO_2_. H_2_O_2_-induced morphological alterations were monitored using an inverted microscope, and the effect of oxidative stress on cell viability was subsequently evaluated using the WST-8 assay [29]. Following H_2_O_2_ exposure, the culture medium was removed and replaced with fresh DMEM/F-12 containing 10% water-soluble 2-(2-methoxy-4-nitrophenyl)-3-(4-nitrophenyl)-5-(2,4-disulfophenyl)-2H-tetrazolium salt-8 (WST-8; Sigma-Aldrich, Catalog Number: 96992, St. Louis, MO, USA). Cells were incubated under standard culture conditions in the dark for 3 h. Wells containing WST-8 reagent without cells were used as blanks for background correction. Absorbance was measured at 450 nm using a microplate reader (VersaMax, Molecular Devices, San Jose, CA, USA), and cell viability was calculated relative to the untreated control group. The H_2_O_2_ concentration that reduced cell viability by approximately 50% while maintaining a sufficient viable cell population for subsequent experiments was selected for establishment of the oxidative stress model. Based on these criteria, 1.5 mM H_2_O_2_ was used in all subsequent experiments. Cell viability was calculated according to the following formula: Cell viability (%) = 100 × (absorbance of the treated group/absorbance of the control group). All experiments were performed in triplicate across three independent experiments.

### 2.3. Isolation and Quantification of hWJ-MSCs-Derived Mitochondria (hWJ-MSCs-Mito)

Mitochondrial isolation was performed using hWJ-MSCs. Briefly, 1 × 10^6^ cells were collected into sterile Eppendorf tubes and centrifuged at 300× *g* for 15 min. Following centrifugation, the supernatant was carefully removed, and the resulting cell pellet was resuspended and homogenized using Standard Hydrogen Electrode (SHE) buffer composed of 0.25 M sucrose, 20 mM HEPES (pH 7.4), 2 mM EGTA, 10 mM KCl, 1.5 mM MgCl_2_, and 0.1% bovine serum albumin (BSA). Homogenization was performed using an insulin syringe to facilitate cellular disruption while preserving mitochondrial integrity. To remove cellular debris, the homogenate was centrifuged at 1100× *g* for 3 min at 4 °C. The resulting supernatant was transferred to a new sterile Eppendorf tube and centrifuged at 1200× *g* for 15 min at 4 °C to pellet the mitochondria. The isolated hWJ-MSCs-mito was subsequently resuspended in 500 μL of SHE buffer and homogenized again to obtain a uniform mitochondrial suspension. Mitochondrial protein content was quantified using the bicinchoninic acid (BCA) method with a SMART BCA Protein Assay Kit (Intron Biotechnology, Catalog Number: 21071, Gyeonggi-do, Republic of Korea) as previously described [29]. Briefly, 0.05% CuSO_4_ was added to a 1% BCA solution, and 5 μL of the mitochondrial suspension was mixed with the reaction buffer and incubated at 50 °C for 30 min. Following incubation, absorbance values were measured spectrophotometrically at 562 nm using a microplate spectrophotometer, and mitochondrial protein concentrations were calculated accordingly. The quantified mitochondrial preparations were subsequently used for transfer experiments.

### 2.4. TMRM Labeling, F-Actin Staining, and Confirmation of Mitochondrial Transfer

To enable visualization and monitoring of mitochondrial transfer, isolated hWJ-MSCs-mito were fluorescently labeled using tetramethylrhodamine methyl ester perchlorate (TMRM; Thermo Fisher Scientific, Catalog Number: M200360, Vilnius, Lithuania) as previously described [30]. TMRM is a lipophilic and cationic fluorescent probe that accumulates within functional mitochondria in a membrane potential-dependent manner. Briefly, mitochondrial suspensions were incubated with 100 nM TMRM at 37 °C for 20 min and subsequently washed with phosphate-buffered saline (PBS) to remove excess dye. COV434 cells were seeded in 24-well culture plates at a density of approximately 15 × 10^3^ cells per well and maintained at 37 °C in a humidified atmosphere containing 5% CO_2_. Following cell attachment, TMRM-labeled hWJ-MSCs-mito suspended in SHE buffer were transferred to COV434 cells. After 24 h, the culture medium was renewed, and cells were further incubated under identical culture conditions for an additional 48 h. To confirm intracellular mitochondrial transfer, cellular boundaries were visualized using an F-actin Staining Kit (Abcam, Catalog Number: ab112125, Cambridge, UK). Briefly, COV434 cells were fixed with 4% paraformaldehyde and permeabilized with 0.1% Triton X-100 (Thermo Fisher Scientific, Catalog Number: A16046.AE, Waltham, MA, USA). Non-specific binding was blocked using PBS containing 1% BSA (Thermo Fisher Scientific, Catalog Number: B14, Waltham, MA, USA) for 10 min. COV434 cells were subsequently incubated with Alexa Fluor-conjugated phalloidin for 30 min at room temperature in the dark to visualize F-actin filaments. Following three PBS washes, fluorescence signals from TMRM-labeled hWJ-MSCs-mito and phalloidin-stained cell boundaries were examined using a confocal laser scanning microscope (Leica TCS SP8, Germany). Images were acquired using standardized imaging parameters and analyzed with LAS X software (Leica Application Suite for Advanced Fluorescence) [31]. The intracellular localization and successful transfer of hWJ-MSCs-mito into COV434 cells were thereby confirmed by confocal microscopy. All fluorescence imaging procedures were performed using identical acquisition settings and microscope parameters [32].

### 2.5. Gene Expression Analysis of Quantitative Real-Time Polymerase Chain Reaction (qRT-PCR)

Total RNA was isolated from COV434 cells subjected to oxidative stress induction and subsequent hWJ-MSCs-mito transfer using the GeneJET RNA Purification Kit (Thermo Fisher Scientific, Catalog Number: K0731, Waltham, MA, USA) according to the manufacturer’s instructions. Briefly, cells were lysed and homogenized, and the resulting lysates were passed through purification columns to remove contaminants and cellular debris. Following a series of washing steps, purified RNA was eluted in RNase-free water. RNA concentration and purity were determined using a NanoDrop spectrophotometer (Thermo Fisher Scientific, Waltham, MA, USA). Complementary DNA (cDNA) was synthesized from 2 μg of total RNA using the High-Capacity cDNA Reverse Transcription Kit (Thermo Fisher Scientific, Catalog Number: 4368814, Waltham, MA, USA). Reverse transcription reactions were carried out in a thermal cycler under the following conditions: 25 °C for 10 min, 37 °C for 120 min, and 85 °C for 5 min, followed by cooling at 4 °C. qRT-PCR analyses were performed using Maxima SYBR Green Master Mix (Thermo Fisher Scientific, Catalog Number: K0253, Waltham, MA, USA) on a LightCycler^®^ 480 II Real-Time PCR System (Roche Diagnostics, Quebec, Canada). Each 20 μL reaction contained 2 μL of cDNA (40 ng), 10 μL of 2× SYBR Green Master Mix, 0.4 μL of forward primer, 0.4 μL of reverse primer, and nuclease-free water. Amplification was performed using an initial denaturation step at 95 °C for 10 min, followed by 45 cycles of 95 °C for 20 s, 60 °C for 30 s, and 72 °C for 30 s. A melt curve analysis ranging from 55 °C to 95 °C was conducted to verify amplification specificity. Relative gene expression levels were calculated using the 2^−ΔΔCt^ method and normalized to β-actin, which served as the endogenous control. Primers targeting cytochrome P450 family 19 subfamily A member 1 (*CYP19A1*), glutathione peroxidase 1 (*GPX1*), heme oxygenase 1 (*HO1*), superoxide dismutase 1 (*SOD1*), and translocase of outer mitochondrial membrane 20 (*TOMM20*) were used to evaluate steroidogenic activity, antioxidant defense, oxidative stress response, and mitochondrial status in COV434 cells following hWJ-MSCs-mito transfer. Primer sequences are presented in Table 1. All reactions were performed in triplicate in three independent experiments.

### 2.6. Immunofluorescence Staining and Evaluation of Caspase-3 and PCNA Markers

For immunofluorescence staining, COV434 cells were exposed to H_2_O_2_-induced oxidative stress and subsequently subjected to hWJ-MSCs-mito transfer. Following the experimental procedures, cells were fixed with 4% paraformaldehyde and permeabilized using 0.1% Triton X-100 (Thermo Fisher Scientific, Catalog Number: A16046.AE, Waltham, MA, USA). To minimize non-specific antibody binding, samples were blocked with PBS containing 1% bovine serum albumin (BSA; Thermo Fisher Scientific, Catalog Number: B14, Waltham, MA, USA) for 30 min at room temperature. Cells were then incubated overnight at 4 °C with an anti-active caspase-3 primary antibody (Active Caspase-3 Rabbit mAb; ABclonal, Catalog Number: A11021, Woburn, MA, USA) and anti-PCNA primary antibody (Elabscience, Catalog Number: E-AB-22001, Houston, TX, USA) at the recommended dilution. Following PBS washes, the appropriate fluorophore-conjugated secondary antibody (ABclonal, Catalog Number: AS070, Woburn, MA, USA) was applied for 2 h at room temperature in the dark. Nuclear counterstaining was performed using 4′,6-diamidino-2-phenylindole (DAPI; Santa Cruz Biotechnology, Dallas, TX, USA). Fluorescence signals were visualized using a confocal laser scanning microscope (Leica TCS SP8, Germany), and images were analyzed using LAS X software (Leica Application Suite for Advanced Fluorescence) [32].

### 2.7. Statistical Analysis

Statistical analyses were conducted using IBM SPSS Statistics version 26.0 (IBM Corp., Armonk, NY, USA). Quantitative data are presented as mean ± standard error of the mean (SEM). The distribution of the data was assessed using the Shapiro–Wilk test to verify normality assumptions. Comparisons among groups were performed using one-way analysis of variance (ANOVA), followed by Tukey’s multiple comparison test for pairwise post hoc analyses. Relative gene expression levels were calculated using the 2^−ΔΔCt^ method prior to statistical evaluation. All quantitative outcomes, including cell viability and gene expression analyses, were statistically analyzed. Each experiment was performed in triplicate and independently repeated at least three times to ensure reproducibility. Statistical significance was defined as *p* < 0.05. In graphical representations, * *p* < 0.05, ** *p* < 0.01, *** *p* < 0.001, and *** *p* < 0.0001 were considered indicative of statistical significance.

## 3. Results

**Figure 1:**
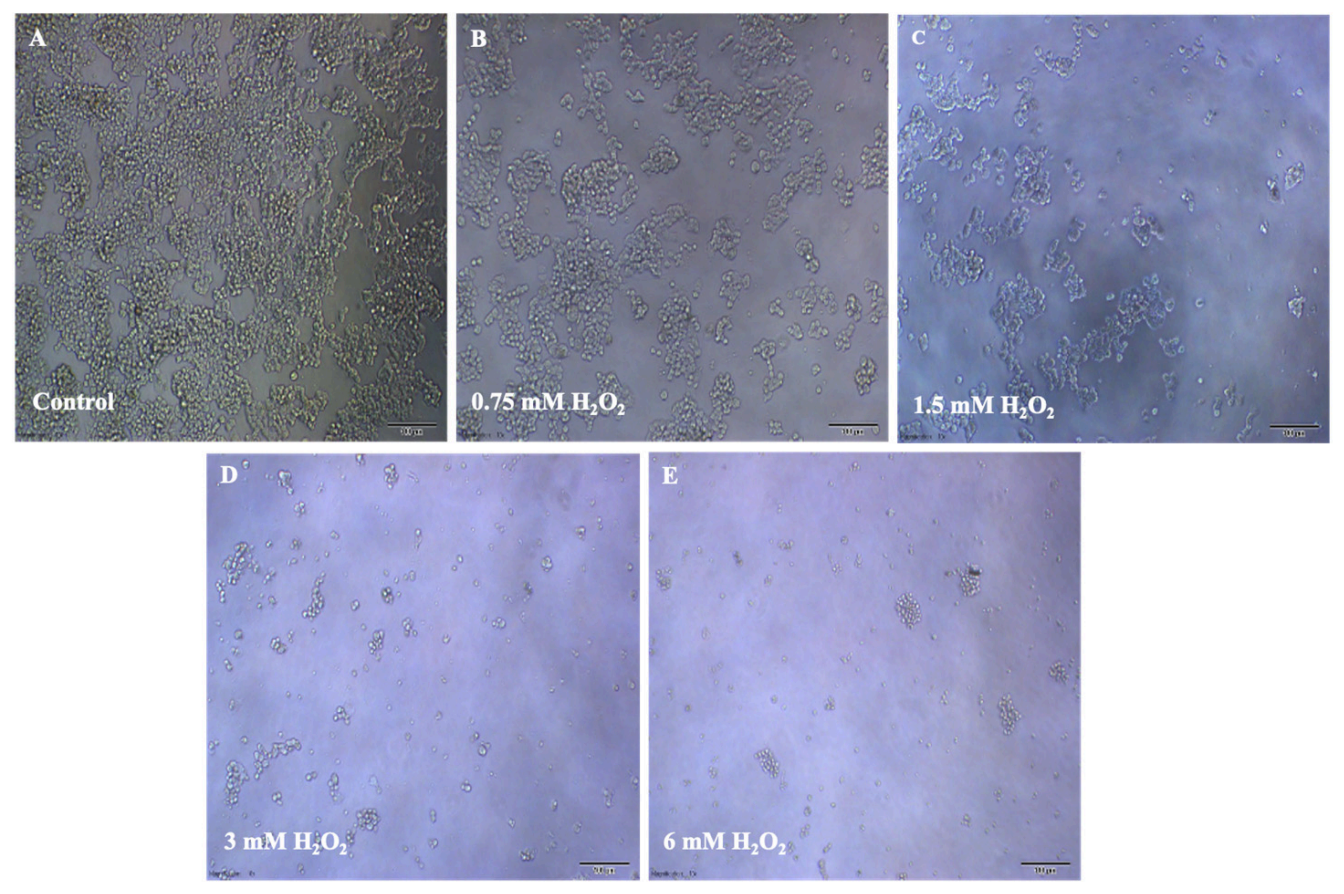
Morphological alterations in COV434 cells following exposure to increasing concentrations of H_2_O_2_. Representative phase-contrast micrographs showing the morphological characteristics of COV434 cells after oxidative stress induction with different H_2_O_2_ concentrations. (**A**) Control, (**B**) 0.75 mM H_2_O_2_, (**C**) 1.5 mM H_2_O_2_, (**D**) 3 mM H_2_O_2_, and (**E**) 6 mM H_2_O_2_. A progressive reduction in cell density and cellular adherence was observed with increasing H_2_O_2_ concentrations, accompanied by marked morphological deterioration at higher doses. Scale bar: 100 μm.

Representative phase-contrast images of COV434 cells following exposure to increasing concentrations of H_2_O_2_ are shown in Figure 1. Control cells (Figure 1A) displayed a dense and uniform monolayer characterized by typical adherent morphology and extensive cell-to-cell contact. Cells exposed to 0.75 mM (Figure 1B) and 1.5 mM H_2_O_2_ (Figure 1C) exhibited a noticeable decrease in cell number and confluency; however, a substantial proportion of the cell population remained attached to the culture surface and retained their overall cellular architecture. In contrast, treatment with 3 mM H_2_O_2_ (Figure 1D) resulted in a marked decline in cell density, accompanied by prominent morphological deterioration and loss of cellular organization. At the highest concentrations examined, 6 mM (Figure 1E), only a limited number of scattered cells and small cellular aggregates were detectable, indicating extensive cellular damage and a pronounced reduction in viable cell populations. Overall, the morphological observations demonstrated a progressive deterioration of cellular integrity with increasing H_2_O_2_ concentrations and were consistent with the corresponding cell viability findings.

**Figure 2:**
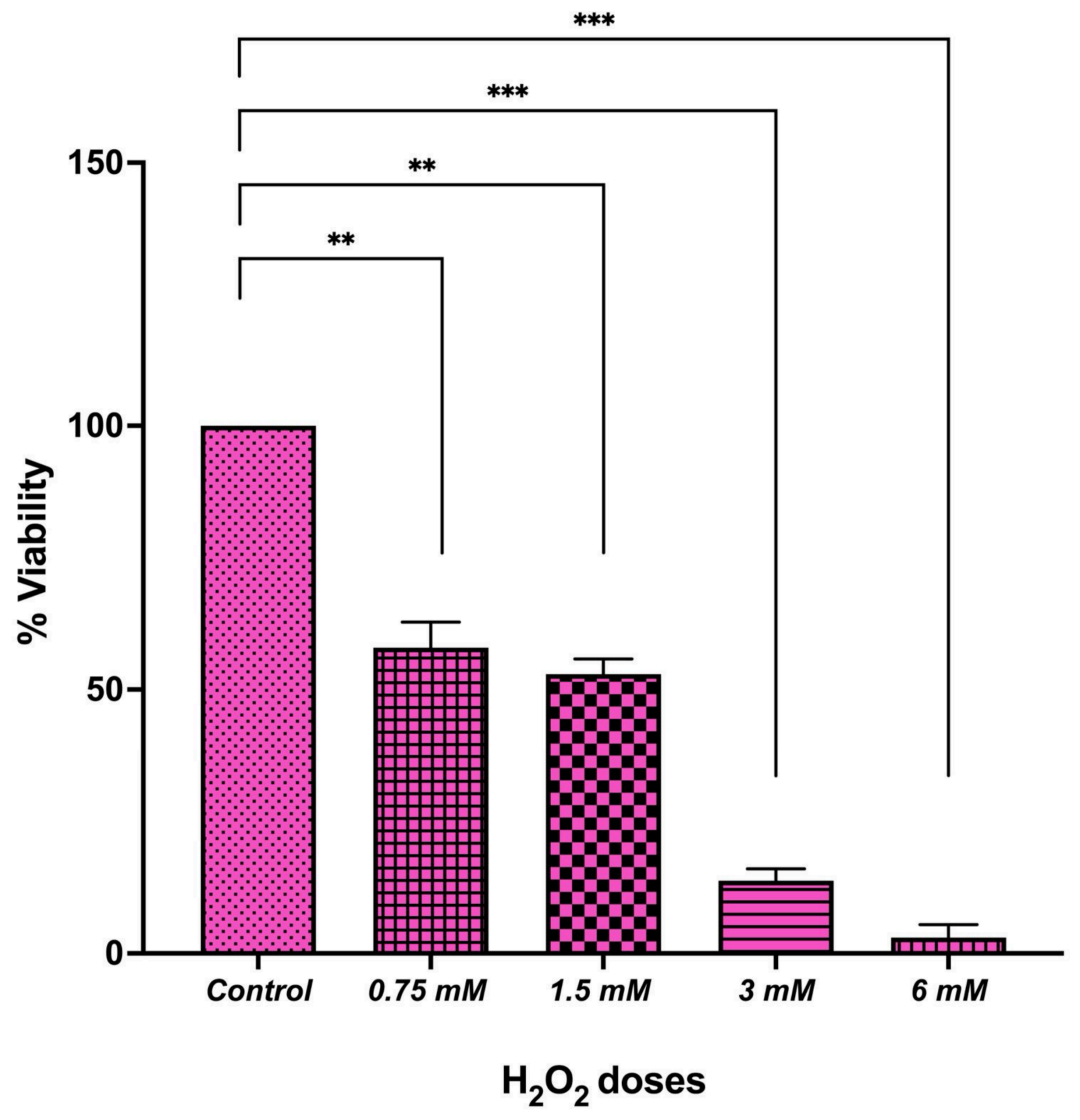
Effects of increasing H_2_O_2_ concentrations on the viability of COV434 cells. COV434 cells were exposed to 0.75, 1.5, 3, or 6 mM H_2_O_2_ for 6 h, and cell viability was determined using the WST-8 assay. Cell viability was normalized to the untreated control group and expressed as a percentage of the control. Data are presented as mean ± SD from three independent experiments. The significance levels are indicated in the graph (** *p* < 0.01, and *** *p* < 0.001).

To establish an oxidative stress model in COV434 cells, the effect of increasing concentrations of H_2_O_2_ on cell viability was evaluated using the WST-8 assay. As shown in Figure 2, H_2_O_2_ exposure resulted in a progressive decline in cell viability in a concentration-dependent manner. While the control group maintained 100% viability, treatment with 0.75 mM and 1.5 mM H_2_O_2_ reduced cell viability to approximately 57% and 52%, respectively. These findings indicate that exposure to these concentrations induced substantial cellular stress while preserving a considerable proportion of the cell population. In contrast, higher H_2_O_2_ concentrations were associated with a pronounced loss of cell viability. Exposure to 3 mM H_2_O_2_ reduced viability to approximately 13%, whereas treatment with 6 mM resulted in near-complete loss of viable cells, reflecting severe cytotoxic effects. Statistical analysis demonstrated significant reductions in cell viability in all H_2_O_2_-treated groups compared with the control group. The most prominent decreases were observed at concentrations of 3 mM and above, where the differences reached a high level of statistical significance (*p* < 0.001). Notably, treatment with 1.5 mM H_2_O_2_ reduced cell viability to nearly half of that observed in the control group. Since this concentration produced a reproducible level of oxidative injury while maintaining sufficient cell survival for subsequent interventions, 1.5 mM H_2_O_2_ was selected as the optimal concentration for the establishment of the oxidative stress model and was therefore used in all subsequent experiments.

**Figure 3:**
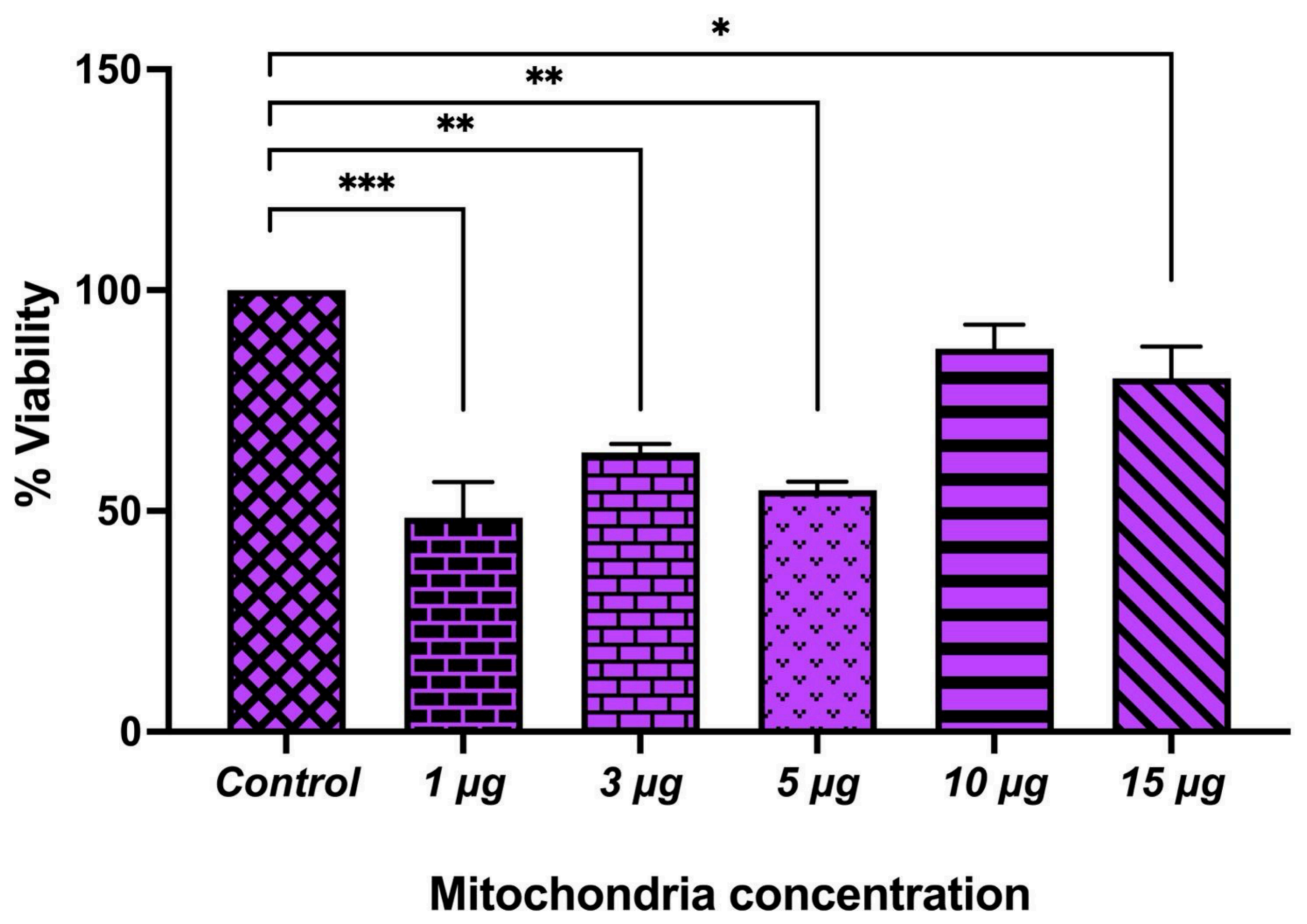
Effects of different concentrations of hWJ-MSC-derived mitochondria on the viability of COV434 cells. These cells were treated with different amounts of hWJ-MSC-derived mitochondria (1, 3, 5, 10, and 15 µg) in the absence of H_2_O_2,_ and cell viability was assessed using the WST-8 assay to determine an appropriate and well-tolerated working amount for subsequent experiments. Cell viability was normalized to the untreated control group and expressed as a percentage of the control. Data are presented as mean ± SD from three independent experiments. The significance levels are indicated in the graph (* *p* < 0.05, ** *p* < 0.01, and *** *p* < 0.001).

The effects of different mitochondrial amounts on COV434 cell viability were evaluated to identify an appropriate mitochondrial dose for subsequent transfer experiments (Figure 3). Cell viability varied across the tested mitochondrial amounts (1, 3, 5, 10, and 15 µg), without exhibiting a monotonic dose-dependent pattern. Compared with the control group, treatment with 1, 3, and 5 µg of mitochondria resulted in significantly lower cell viability (*p* < 0.05–0.001). Among the tested mitochondrial amounts, 10 µg resulted in the highest cell viability, reaching approximately 86% of the control value. The difference between the 10 µg group and the untreated control did not reach statistical significance (*p* = 0.059), indicating that this mitochondrial amount was well tolerated by COV434 cells under the experimental conditions. Increasing the mitochondrial amount to 15 µg did not result in a further improvement in cell viability, with viability remaining lower than that observed in the 10 µg group. Based on its favorable viability profile and cellular tolerability, 10 µg was selected as the working mitochondrial dose for subsequent transfer experiments.

**Figure 4:**
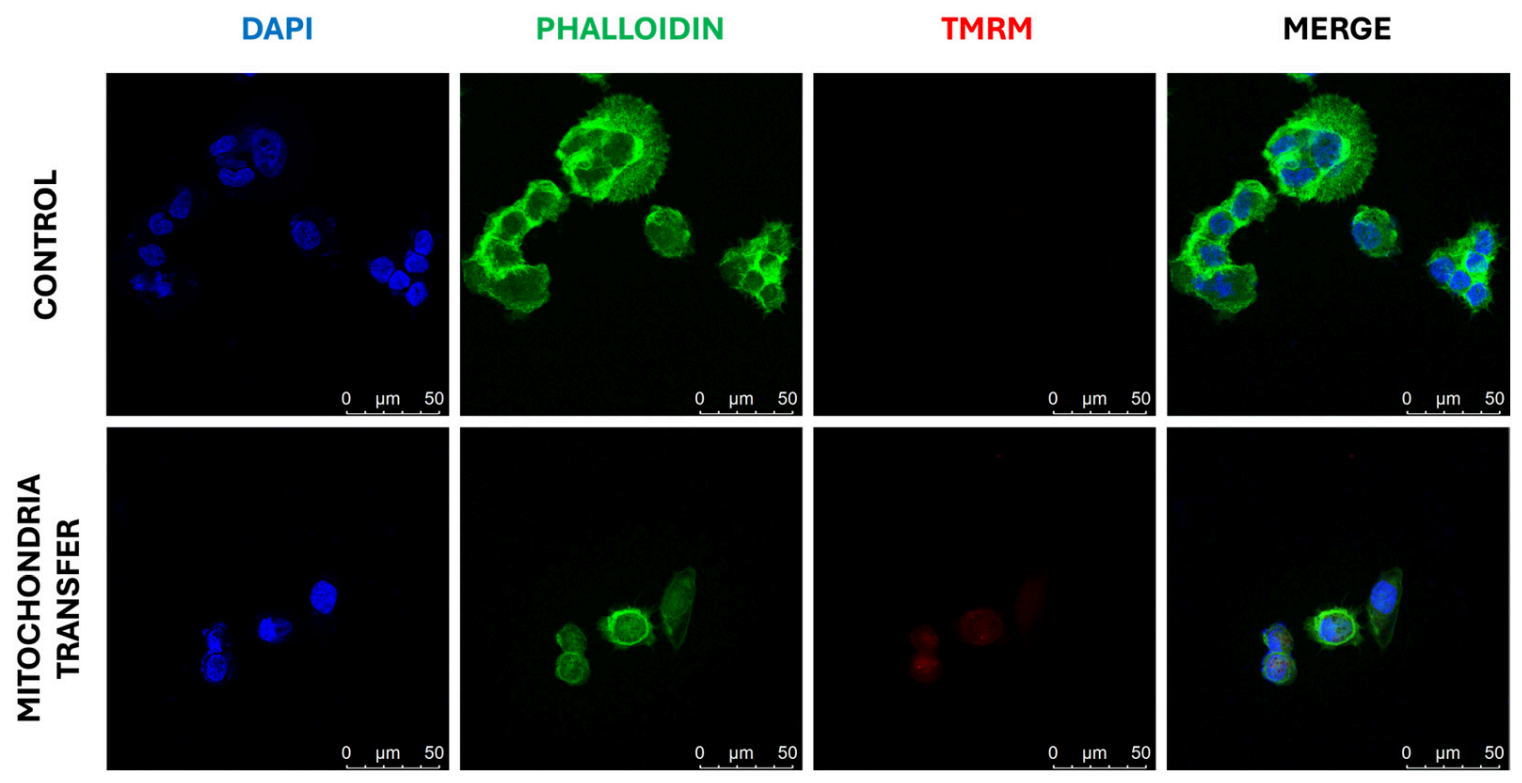
Representative confocal images showing the uptake of TMRM-labeled hWJ-MSC-derived mitochondria by COV434 cells. Cell nuclei were stained with DAPI (blue), F-actin was labeled with phalloidin (green), and transferred mitochondria were visualized with TMRM (red). Merged images demonstrate the intracellular localization of TMRM-positive mitochondria within recipient COV434 cells. Scale bar = 50 µm.

Representative confocal images demonstrated the intracellular localization of TMRM-labeled hWJ-MSC-derived mitochondria in COV434 cells. In the control group, no TMRM fluorescence was detected. In contrast, distinct intracellular TMRM-positive signals were observed following mitochondrial treatment within the phalloidin-defined cell boundaries, indicating the successful uptake of exogenous mitochondria by recipient COV434 cells (Figure 4).

**Figure 5:**
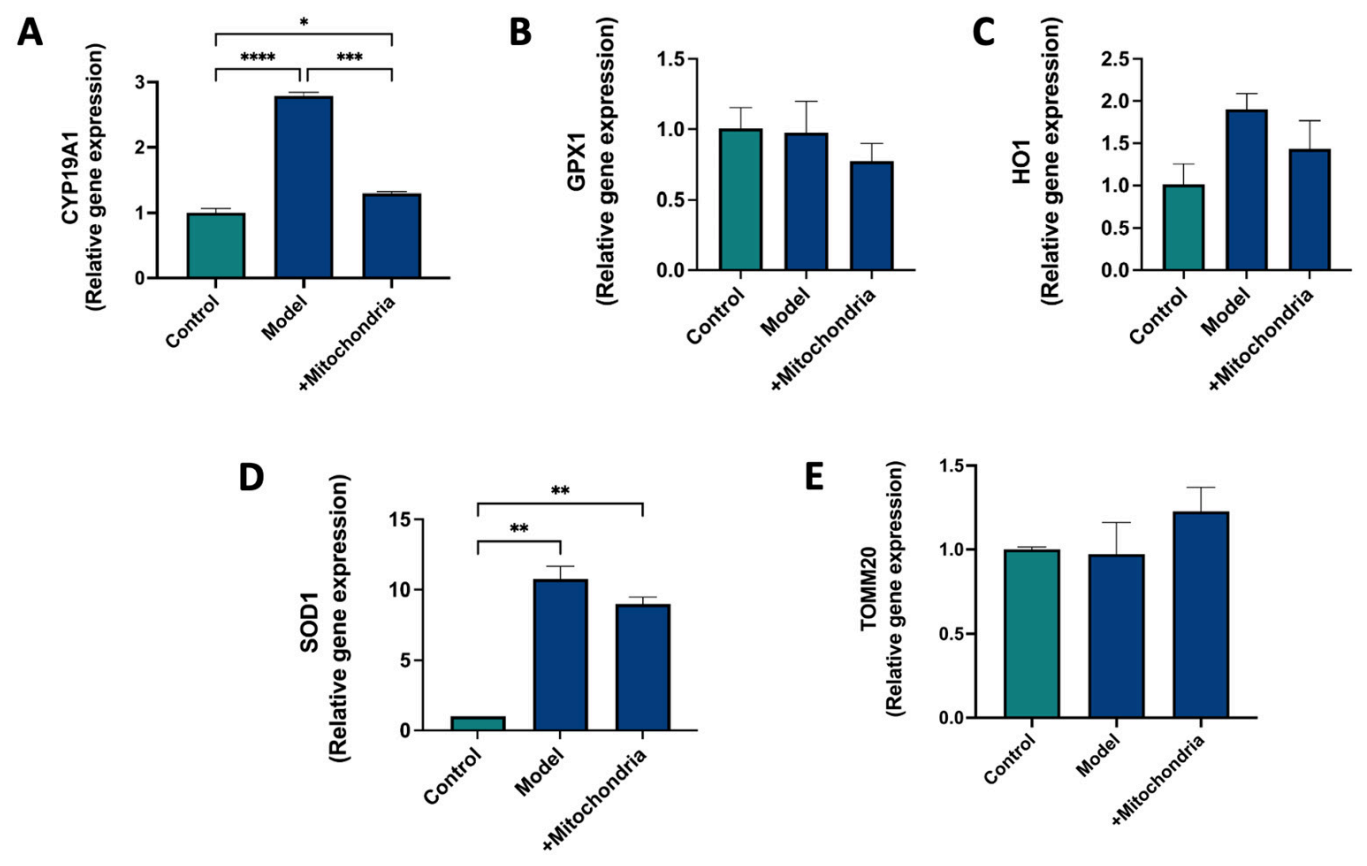
Effects of hWJ-MSCs-mito transfer on the expression of oxidative stress-, steroidogenesis, and mitochondria-related genes in H_2_O_2_-treated COV434 cells. Relative mRNA expression levels of (**A**) CYP19A1, (**B**) GPX1, (**C**) HO1, (**D**) SOD1, and (**E**) TOMM20 were determined by qRT-PCR and normalized to β-actin. Gene expression levels were calculated using the 2-ΔΔCt method and are presented relative to the control group. Control: untreated cells; Model: H_2_O_2_-treated cells; +mit: H_2_O_2_-treated cells receiving hWJ-MSCs-mito transfer. Data are presented as mean ± SEM. Statistical significance is indicated as * *p* < 0.05, ** *p* < 0.01, *** *p* < 0.001, **** *p* < 0.0001.

Gene expression analyses were performed using the 10 μg mitochondrial dose selected based on the preceding cell-viability assessment. *CYP19A1* expression was significantly increased in the H_2_O_2_-treated model group compared with the control group, reaching approximately 2.8-fold higher levels (Figure 5A). Following hWJ-MSCs-mito transfer, *CYP19A1* expression was markedly reduced and was restored toward control values. Although expression levels remained slightly elevated relative to the control group, a substantial decrease was observed compared with the model group. These findings indicated that *CYP19A1* transcription was markedly altered under oxidative stress conditions and was partially normalized following mitochondrial transfer. As *CYP19A1* encodes aromatase, a key enzyme involved in estradiol biosynthesis in granulosa cells, the observed changes were considered to reflect alterations in steroidogenesis-related transcription. The reduction in *CYP19A1* expression following mitochondrial transfer indicated partial modulation of the H_2_O_2_-associated transcriptional response toward the control profile, rather than complete normalization of *CYP19A1* expression. *GPX1* expression was found to be relatively stable across all experimental groups (Figure 5B). Expression levels measured in the model group were comparable to those detected in the control group, whereas a slight decrease was observed following hWJ-MSCs-mito transfer. However, the magnitude of this change remained limited. These findings suggested that *GPX1* transcription was not substantially affected by H_2_O_2_ exposure under the experimental conditions employed in this study. As *GPX1* functions as a major glutathione-dependent antioxidant enzyme involved in hydrogen peroxide detoxification, its stable expression profile was considered indicative of preserved basal antioxidant defense activity. The absence of marked transcriptional alterations suggested that *GPX1* was not among the principal antioxidant pathways modulated by oxidative stress or mitochondrial transfer. *HO1* expression did not differ significantly among the experimental groups (Figure 5C). Although variation in HO1 expression was observed across the control, model, and hWJ-MSCs-mito groups, none of the between-group comparisons reached statistical significance. Therefore, the observed expression pattern does not indicate a significant effect of either H_2_O_2_ exposure or hWJ-MSCs-mito transfer on HO1 expression under the experimental conditions evaluated. *HO1* is a stress-responsive gene involved in cellular defense against oxidative challenge, and its expression pattern in the present study showed a tendency to increase following H_2_O_2_ exposure and subsequently decrease following mitochondrial supplementation. Given the absence of statistical significance, however, these changes indicate a transcriptional tendency rather than evidence of normalization of the oxidative stress response.

*SOD1* expression was markedly elevated in both the model and hWJ-MSCs-mito groups compared with the control group (Figure 5D). The highest expression level was detected in the model group, with an approximately 10-fold increase relative to baseline. *SOD1* expression remained significantly elevated in both the model and mitochondrial treatment groups compared with the control group. However, no statistically significant difference in *SOD1* expression was observed between the model and hWJ-MSCs-mito groups, indicating that mitochondrial transfer did not significantly alter the H_2_O_2_-associated *SOD1* expression under the experimental conditions evaluated. Thus, the oxidative stress-associated *SOD1* transcriptional response persisted following mitochondrial supplementation. Only minor variations were observed in *TOMM20* expression among the experimental groups (Figure 5E). Expression levels detected in the model group were comparable to those measured in the control group, whereas a modest increase was observed following hWJ-MSCs-mito transfer. Although *TOMM20* expression varied among the experimental groups, the differences did not reach statistical significance. Therefore, no significant change in *TOMM20* expression was observed following H_2_O_2_ exposure or mitochondrial supplementation, and the findings do not indicate a significant treatment-related effect on *TOMM20* expression or mitochondrial status.

**Figure 6:**
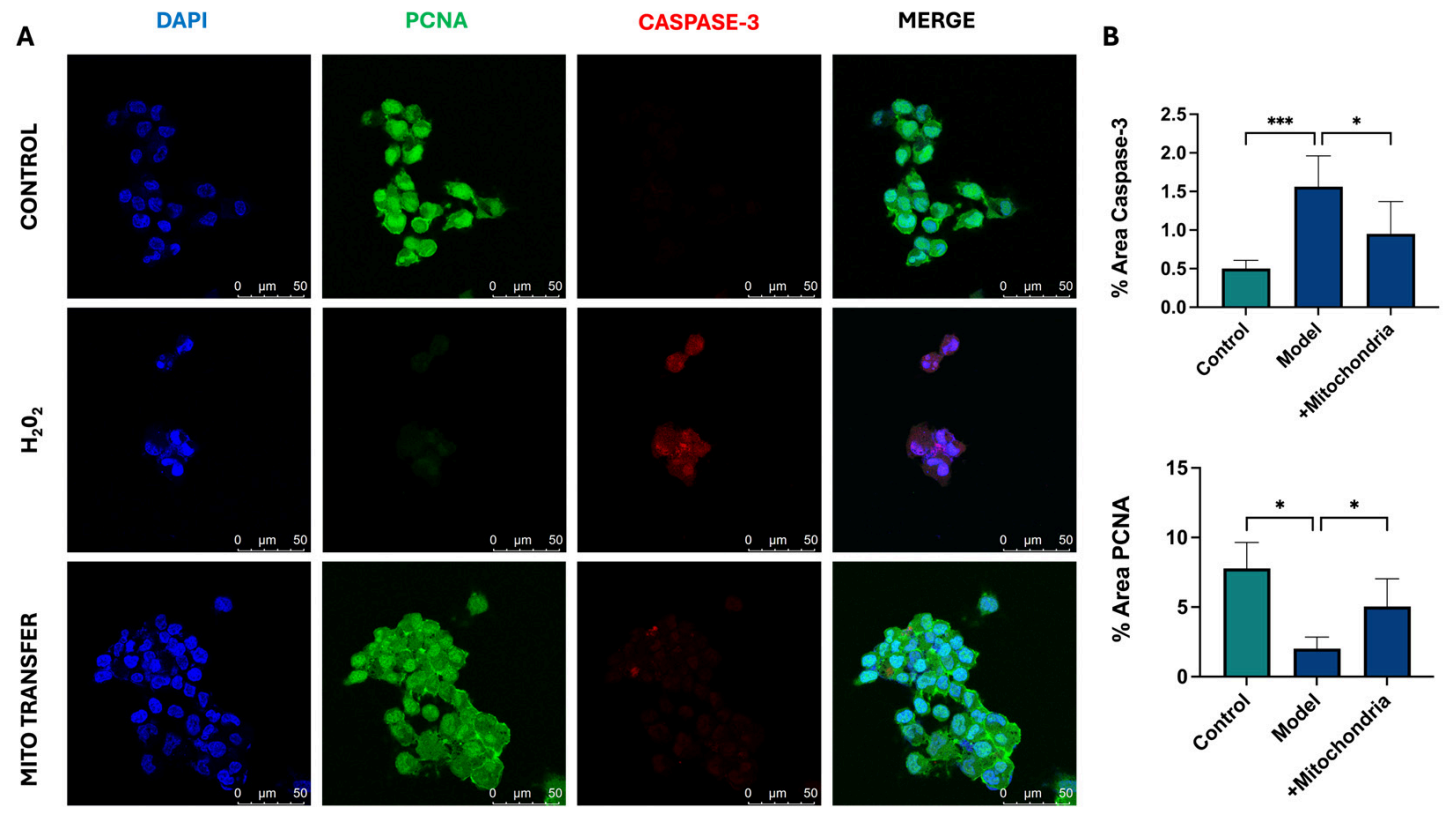
Representative immunofluorescence images and quantitative analysis of PCNA and active caspase-3 expression in COV434 cells. DAPI (blue) was used for nuclear staining, PCNA (green) for proliferative cells, and active caspase-3 (red) for apoptotic cells. Representative images are shown for the Control, H_2_O_2_, and H_2_O_2_ + hWJ-MSC-derived mitochondria groups (**A**). Quantitative analysis of the PCNA-positive area showed a significant decrease in the H_2_O_2_ group compared with the Control group and a significant increase following mitochondrial supplementation compared with the H_2_O_2_ group (*p* < 0.05). Quantitative analysis of the active caspase-3-positive area showed a significant increase in the H_2_O_2_ group compared with the Control group (*p* < 0.0001) and a significant decrease following mitochondrial supplementation compared with the H_2_O_2_ group (*p* < 0.05) (**B**). Scale bar = 50 µm. The significance levels are indicated in the graph (* *p* < 0.05 and *** *p* < 0.001).

Representative immunofluorescence images demonstrated distinct differences in PCNA and active caspase-3 immunoreactivities among the experimental groups. Control cells exhibited strong PCNA immunoreactivity with weak active caspase-3 staining, indicating preserved proliferative activity and low basal apoptotic signaling. H_2_O_2_ treatment was associated with decreased PCNA immunoreactivity and increased active caspase-3 immunoreactivity, consistent with reduced cellular proliferation and enhanced apoptosis under oxidative stress (Figure 6A). Quantitative analysis showed that H_2_O_2_ exposure significantly reduced the PCNA-positive area from 7.784% in the control group to 2.343% in the model group (*p* < 0.05). Following mitochondrial supplementation, the PCNA-positive area significantly increased to 5.041% compared with the H_2_O_2_-treated model group (*p* < 0.05). Similarly, H_2_O_2_ exposure significantly increased the active caspase-3-positive area from 0.500% in the control group to 1.561% in the model group (*p* < 0.0001), whereas mitochondrial supplementation significantly decreased the active caspase-3-positive area to 0.950% compared with the H_2_O_2_-treated model group (*p* < 0.05) (Figure 6B). In contrast, cells receiving hWJ-MSC-derived mitochondrial treatment showed higher PCNA immunoreactivity and lower active caspase-3 immunoreactivity than the H_2_O_2_-treated group, suggesting partial preservation of proliferative activity together with attenuation of apoptotic responses (Figure 6A).

## 4. Discussion

Our investigation showed that hWJ-MSC-derived mitochondrial transfer was associated with reduced oxidative stress-induced injury in COV434 granulosa cells, as reflected by improved cell survival, reduced apoptotic signaling, and modulation of selected oxidative stress- and mitochondria-related molecular responses. Successful internalization of exogenous mitochondria was accompanied by increased proliferative activity, decreased active caspase-3 immunoreactivity, attenuation of oxidative stress-responsive gene expression, and partial modulation of *CYP19A1* expression toward physiological levels, suggesting a favorable cellular response to mitochondrial supplementation. Since mitochondrial dysfunction is considered a major contributor to granulosa cell apoptosis, follicular atresia, and impaired female fertility [10,33], these findings indicate that mitochondrial supplementation may contribute to cellular recovery following oxidative injury rather than merely replace damaged organelles. Although the therapeutic effects of mesenchymal stem cells have largely been attributed to their paracrine activity [27], our findings further support the concept that direct mitochondrial transfer may contribute to the cellular response of granulosa cells exposed to oxidative injury. Overall, this study expands the current understanding of mitochondrial supplementation in reproductive biology and provides experimental evidence supporting further investigation of mitochondrial transfer in ovarian cells exposed to oxidative stress.

The successful establishment of the oxidative stress model represents a critical prerequisite for evaluating the therapeutic efficacy of mitochondrial transfer. In the present study, H_2_O_2_ exposure induced a marked reduction in COV434 cell viability accompanied by progressive morphological deterioration, confirming the development of oxidative injury in a concentration-dependent manner. These findings are consistent with previous studies demonstrating that H_2_O_2_ is one of the most widely used in vitro oxidative stress inducers in granulosa cells because of its ability to rapidly diffuse across cellular membranes, increase intracellular reactive oxygen species (ROS) levels, and trigger mitochondrial dysfunction and apoptotic signaling [7,9]. Excessive ROS accumulation disrupts mitochondrial membrane integrity, impairs ATP production, and activates intrinsic apoptotic pathways, ultimately compromising granulosa cell survival and follicular development [8,10]. Consistent with these mechanisms, the approximately 50% reduction in cell viability observed following 1.5 mM H_2_O_2_ exposure indicated that our experimental model generated sufficient oxidative damage while preserving an adequate population of viable cells for subsequent therapeutic intervention. This intermediate level of injury is considered particularly appropriate for evaluating regenerative approaches because excessively severe oxidative damage may obscure potential treatment-related benefits. Therefore, the oxidative stress model established in the present study provided a biologically relevant experimental platform for investigating the protective effects of hWJ-MSC-derived mitochondrial transfer under conditions that closely resemble oxidative damage occurring during ovarian aging and other oxidative stress-associated reproductive disorders.

Efficient intracellular delivery of exogenous mitochondria is a fundamental prerequisite for mitochondrial transplantation to produce a biological effect. The confocal observations presented in Figure 4 confirmed that TMRM-labeled hWJ-MSC-derived mitochondria were detected within the phalloidin-defined boundaries of COV434 granulosa cells, supporting the successful uptake of isolated mitochondria by recipient cells. Although the mechanisms responsible for extracellular mitochondrial uptake have not been fully elucidated, experimental evidence indicates that isolated mitochondria can enter recipient cells through actin-dependent endocytic processes, including macropinocytosis, with the predominant route varying according to the recipient cell type and experimental conditions [34,35]. Nevertheless, intracellular detection alone does not establish the functional integration or metabolic competence of transferred mitochondria. The accompanying changes in cell viability, proliferation- and apoptosis-related immunoreactivity, and stress-responsive gene expression indicated that mitochondrial administration was associated with a broader cellular response following oxidative injury (Figure 5 and Figure 6). Previous studies have reported that transferred mitochondria may improve cellular bioenergetics and support recovery in damaged recipient cells [36,37]; however, direct assessment of ATP generation, respiratory activity, mitochondrial membrane potential, and integration into the endogenous mitochondrial network would be required to confirm these mechanisms in COV434 cells. Thus, the findings presented here establish successful mitochondrial uptake and provide a basis for examining whether the internalized organelles contribute directly to the functional recovery of oxidatively stressed granulosa cells.

An important finding emerging from this study was the improvement in granulosa cell viability and proliferative activity following hWJ-MSC-derived mitochondrial transfer. Mitochondria significantly enhanced cell viability while partially restoring PCNA immunoreactivity in H_2_O_2_-exposed COV434 cells, indicating that mitochondrial transfer alleviated, at least in part, the detrimental effects of oxidative stress on granulosa cell survival and proliferative capacity (Figure 3 and Figure 6). Because granulosa cell proliferation is indispensable for follicular growth, steroidogenesis, and the maintenance of oocyte developmental competence, disruption of mitochondrial homeostasis has been recognized as a critical event leading to impaired follicular development under oxidative stress conditions [38]. In agreement with previous studies, restoration of mitochondrial integrity has been associated with improved cellular bioenergetics and enhanced regenerative capacity in injured recipient cells following mitochondrial transfer [36]. Although mitochondrial respiration and ATP production were not directly evaluated in our experimental model, the concomitant increase in WST-8-derived cell viability and PCNA immunoreactivity indicates a favorable cellular response to mitochondrial supplementation under oxidative stress. Importantly, this improvement in cell viability occurred without significant treatment-related changes in *GPX1*, *HO1*, *SOD1*, or *TOMM20* expression, suggesting that the observed cellular response was not mediated through transcriptional regulation of these selected genes. The accompanying increase in PCNA and decrease in active caspase-3 immunoreactivity suggest that enhanced proliferative activity and attenuation of apoptotic signaling may contribute to the observed improvement in cell viability, although the underlying molecular mechanism cannot be established from the present findings.

Another notable finding was the marked reduction in active caspase-3 immunoreactivity following hWJ-MSC-derived mitochondrial supplementation in H_2_O_2_-treated COV434 cells (Figure 6), suggesting attenuation of the apoptotic response induced by oxidative stress. Caspase-3 is a principal executioner caspase activated during the execution phase of apoptosis following upstream apoptotic signaling. Disruption of mitochondrial homeostasis under oxidative stress can promote apoptotic signaling and ultimately contribute to granulosa cell apoptosis and follicular atresia. Recent evidence has further highlighted mitochondrial dysfunction and excessive oxidative stress as important contributors to granulosa cell loss through enhanced apoptotic signaling and impaired follicular integrity [10]. In the present study, the reduction in active caspase-3 immunoreactivity following mitochondrial treatment indicates attenuation of active caspase-3-associated apoptotic signaling. This finding is consistent with previous studies showing that attenuation of oxidative stress in H_2_O_2_-induced granulosa cell models is accompanied by reduced apoptotic signaling and improved cell survival [39]. Accordingly, the present findings support an association between mitochondrial supplementation and reduced active caspase-3 immunoreactivity under oxidative stress, while the molecular events preceding caspase-3 activation remain to be further elucidated.

Beyond the attenuation of apoptotic signaling, mitochondrial supplementation was also associated with distinct transcriptional responses in antioxidant defense-related genes (Figure 5B–D). *GPX1*, *HO1*, and *SOD1* exhibited different expression patterns following oxidative injury and subsequent mitochondrial treatment, indicating that antioxidant-related genes were not uniformly regulated under the experimental conditions employed. *GPX1* expression remained relatively stable across all experimental groups, with no statistically significant differences, suggesting that *GPX1* transcription was not substantially affected by either H_2_O_2_ exposure or mitochondrial supplementation. *HO1* expression did not differ significantly among the experimental groups following H_2_O_2_ exposure and mitochondrial supplementation; therefore, no significant treatment-related change in *HO1* expression was observed. Because *HO1* is an inducible cytoprotective enzyme that is rapidly activated under conditions of oxidative stress, the observed expression pattern may reflect a stress-responsive transcriptional tendency; nevertheless, in the absence of statistical significance, it should not be interpreted as evidence of normalization of the intracellular oxidative environment [40]. A different expression pattern was observed for *SOD1*, which was markedly increased following H_2_O_2_ exposure and remained significantly elevated compared with the control group after mitochondrial administration. *SOD1* expression did not differ significantly between the mitochondrial treatment group and the model group, indicating persistence of the oxidative stress-associated *SOD1* transcriptional response following mitochondrial supplementation. Similar gene-specific regulation of antioxidant defense genes has been reported in oxidatively stressed granulosa cells, where the transcriptional responses of individual antioxidant genes varied according to the severity of oxidative injury and the stage of cellular adaptation [41]. Since intracellular ROS accumulation, glutathione homeostasis, NRF2 activation, and antioxidant enzyme activities were not evaluated, the molecular mechanisms underlying these differential transcriptional responses could not be fully elucidated. Taken together, the expression profiles of *GPX1*, *HO1*, and *SOD1* indicate a heterogeneous antioxidant transcriptional response to H_2_O_2_-induced oxidative stress and mitochondrial supplementation, rather than a uniform normalization of antioxidant defense pathways.

Although *TOMM20* expression did not differ significantly among the experimental groups, a modest upward trend was observed following mitochondrial supplementation (Figure 5E). *TOMM20* is a major component of the translocase of the outer mitochondrial membrane (TOM) complex and plays an essential role in the recognition and import of nuclear-encoded proteins into mitochondria, thereby contributing to the maintenance of mitochondrial integrity and function. Preservation of this import machinery is critical for mitochondrial quality control because most mitochondrial proteins are synthesized in the cytosol and subsequently transported into mitochondria through the TOM complex [42,43]. *TOMM20* expression did not differ significantly among the experimental groups following mitochondrial supplementation; therefore, these findings should not be interpreted as evidence of a significant treatment-related change in mitochondrial integrity or function. Previous studies have demonstrated that preservation of mitochondrial protein import is closely associated with the maintenance of mitochondrial function and cellular adaptation under conditions of oxidative stress [44]. Nevertheless, mitochondrial respiration, membrane potential, ATP production, and mitochondrial ultrastructure were not evaluated in the present study, and it therefore remains unclear whether *TOMM20* expression is associated with any functional alteration in the mitochondrial network. Accordingly, the *TOMM20* findings should be considered supportive but exploratory and interpreted together with the observed changes in cell viability, apoptosis-related signaling, and oxidative stress-related gene expression rather than as an independent indicator of restored mitochondrial function.

The expression pattern of *CYP19A1* provided additional insight into the transcriptional response of granulosa cells to mitochondrial supplementation beyond the observed changes in oxidative stress- and apoptosis-related markers. *CYP19A1* expression was markedly increased following H_2_O_2_ exposure and was significantly reduced after treatment with hWJ-MSC-derived mitochondria, although basal expression levels were not fully restored (Figure 5A). *CYP19A1* encodes aromatase, the rate-limiting enzyme responsible for the conversion of androgens into estrogens within granulosa cells and therefore plays a pivotal role in follicular steroidogenesis, oocyte maturation, and maintenance of normal ovarian function. Alterations in *CYP19A1* expression have been closely associated with changes in estradiol biosynthesis and granulosa cell steroidogenic activity [45]. Interestingly, the marked increase in *CYP19A1* expression observed after H_2_O_2_ exposure differed from the reduction in aromatase expression that has frequently been described under chronic oxidative stress conditions. This discrepancy may reflect the acute nature of the oxidative insult, the biological characteristics of the COV434 granulosa tumor-derived cell line, or adaptive transcriptional responses during the early phase of oxidative injury. Granulosa cell *CYP19A1* expression is known to be highly responsive to context-dependent regulatory pathways, including cAMP/PKA-mediated signaling, and may therefore exhibit distinct transcriptional responses depending on the cellular environment and experimental conditions [46]. Following mitochondrial supplementation, *CYP19A1* expression was significantly reduced compared with the oxidative stress model but remained higher than that of the control group, indicating partial modulation rather than complete normalization of *CYP19A1* transcription. Because aromatase protein abundance, enzymatic activity, estradiol secretion, and other steroidogenic markers were not evaluated, the functional consequences of the observed transcriptional changes could not be determined directly. Therefore, the present findings support an association between mitochondrial supplementation and modulation of the oxidative stress-associated *CYP19A1* transcriptional response but should not be interpreted as direct evidence of restoration of steroidogenic function.

The interpretation of these transcriptional findings should also be considered in the context of the mitochondrial dose-selection strategy used in the present study. Mitochondrial amounts of 1, 3, 5, 10, and 15 μg were initially evaluated using cell viability as the endpoint to identify an appropriate and well-tolerated working dose for subsequent experiments, rather than to establish molecular dose–response relationships. Among the tested amounts, 10 μg resulted in the highest cell viability and did not differ significantly from the untreated control, whereas increasing the mitochondrial amount to 15 μg did not provide an additional improvement in viability. Accordingly, 10 μg was selected as the working dose for subsequent gene expression analyses. Therefore, the changes observed in *CYP19A1*, *GPX1*, *HO1*, *SOD1*, and *TOMM20* represent the transcriptional responses associated with the selected 10 μg mitochondrial dose and should not be interpreted as evidence of dose-dependent regulation of these genes. Evaluation of these molecular endpoints across multiple mitochondrial doses could provide a more detailed characterization of their dose–response relationships; however, such an analysis was beyond the predefined scope of the present experimental design and warrants investigation in future studies.

## 5. Conclusions

The findings of this study suggest that mitochondrial supplementation may represent a promising approach for modulating cellular responses to oxidative stress-induced injury in granulosa cells. Since granulosa cells are essential for maintaining the microenvironment required for follicular development and oocyte maturation, preservation of their viability is important for normal ovarian physiology. Although the present findings were obtained in an in vitro model, the observed changes in cell viability, apoptotic signaling, and *CYP19A1* expression indicate that hWJ-MSC-derived mitochondrial supplementation is associated with favorable cellular responses and modulation of transcriptional responses under oxidative stress conditions. Further studies using primary human granulosa cells and in vivo models will be necessary to determine whether these cellular effects can be translated into reproductive outcomes. Several limitations of this study should be considered when interpreting the findings. The experiments were performed using an in vitro granulosa cell model, and functional mitochondrial parameters, including ATP production and mitochondrial respiration, were not evaluated. Therefore, the mechanisms associated with the observed effects of mitochondrial supplementation remain to be clarified. Despite these limitations, the study combined cellular, immunofluorescence, and gene expression analyses to provide a comprehensive evaluation of the response of granulosa cells to hWJ-MSC-derived mitochondrial supplementation under oxidative stress conditions. Overall, these findings suggest that mitochondrial supplementation is associated with attenuation of selected cellular and molecular alterations induced by oxidative stress, thereby providing a basis for future mechanistic and translational studies.

## Figures and Tables

**Table 1:** Primer base sequences for genes used in qRT-PCR analysis.

Genes	Primer Base Sequence (5′→3′)
*CYP19A1*	**F-** CAGAGTATCCGGAGGTGGAA **R-** CATGACCAAGTCCACGACAG
*GPX1*	**F-** TTGACATCGAGCCTGACATC **R-** ACTGGGATCAACAGGACCAG
*HO1*	**F-** GGTGTCCAGGGAAGGCTTTAAG **R-** GGTGTCCAGGGAAGGCTTTAAG
*SOD1*	**F-** CGGATGAAGAGAGGCATGTT **R-** CACCTTTGCCCAAGTCATCT
*TOMM20*	**F-** AAACAGAAGCTTGCCAAGGA **R-** CATCTGGAACACTGGTGGTG
*β-actin*	**F-** AGAGAAGCTGTGCTATGTTG **R-** GTACTCCTGCTTGCTGATCC

## Data Availability

The data presented in this study are available on request from the corresponding author. The data are not publicly available due to privacy and ethical restrictions.
